# Efficiency and safety of five different agents for *in vivo* delivery of novel bioengineered RNAi molecules

**DOI:** 10.3389/fmolb.2026.1785592

**Published:** 2026-03-13

**Authors:** Su Guan, Mei-Juan Tu, Yan-Ju Li, Yimei Wang, Ai-Ming Yu

**Affiliations:** Department of Biochemistry and Molecular Medicine, School of Medicine, University of California at Davis, Sacramento, CA, United States

**Keywords:** bioengineered RNA, invivofectamine, lipid nanoparticles, RNA delivery, RNA therapeutics, RNAi, siRNA, transgenic mouse

## Abstract

RNA molecules have emerged as an addition to existing entities for therapy and vaccination, whose success may be hindered by inefficient *in vivo* delivery or induction of excessive toxicities, such as severe cytokine release syndrome. In this study, we used a novel, bioengineered RNA (BioRNA) bearing payload siRNA against green fluorescent protein (GFP) (BioRNA/GFP-siRNA) and a GFP-transgenic mouse model to compare the efficiency and safety of five commercial agents, namely lipid nanoparticles (LNP) and Invivofectamine, as well as Nanoparticle, LIPID-, and PEG-Liposome based *In Vivo* Transfection Reagents. The results showed that all products provided effective delivery of BioRNA/GFP-siRNA into mouse livers to elicit RNA interference (RNAi) effects. Among them, the LNP, Invivofectamine, and nanoparticle formulations showed relatively greater efficacy, as manifested by higher siRNA accumulation or lower GFP mRNA levels and fluorescence intensity. However, the MC3-based LNP-BioRNA treatment led to an 8% decrease in body weights and obvious hepatosplenomegaly, as well as statistically significant changes in liver and kidney function biomarkers and elevation of multiple pro-inflammatory cytokines, while all other formulations were generally well tolerated. In addition, delivery efficiency of these *in vivo* transfection agents determined in cells *in vitro* were not proportional to their performance in mice *in vivo*. These findings highlight the differences among these RNA delivery systems examined herein and underscore the importance of rigorous evaluation of both efficacy and safety when selecting appropriate platforms for RNA agents.

## Graphical Abstract

**Figure:**
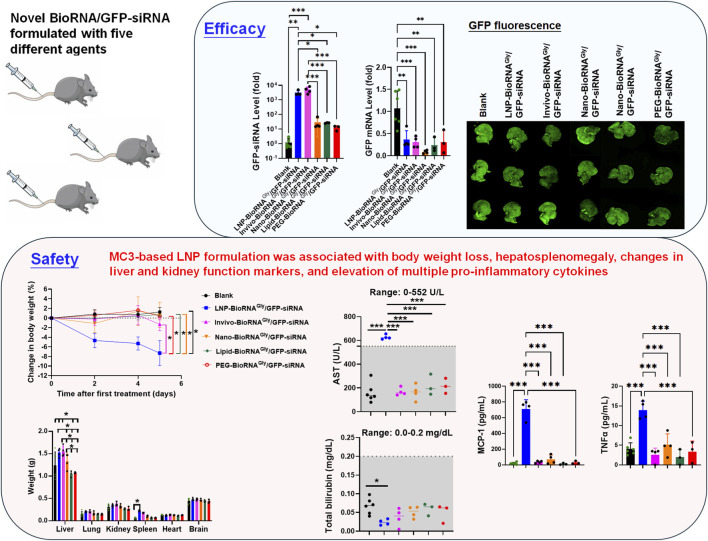

## 1 Introduction

RNA interference (RNAi) has emerged as a powerful approach for therapeutic gene silencing, enabling the selective inhibition of disease-related genes at the post-transcriptional level (Fire et al., 1998; Bartel, 2004; Meister and Tuschl, 2004; Yu et al., 2020; Traber and Yu, 2023; Jadhav et al., 2024; Traber and Yu, 2024). Small interfering RNAs (siRNAs) are common RNAi agents that are short double-stranded RNAs, each containing an antisense and a sense strand about 21–23 nt in length that mirror the guide and passenger strand of genome-derived microRNA (miRNA or miR), respectively. In particular, the guide or antisense strand is the one that executes broad RNAi action through inhibition of translation or induction of mRNA degradation, upon selectively incorporated into the RNA-induced silencing complex (RISC) and complementary Watson-Crick base pairing with targeted transcripts (Traber and Yu, 2024; Cronin and Yu, 2025). While siRNAs typically induce Argonaute 2-mediated endonucleolytic cleavage of perfectly complementary mRNA targets, miRNAs more often regulate target gene expression through translational repression and/or mRNA destabilization involving imperfect base pairing (Hammond et al., 2001; Meister and Tuschl, 2004). A key feature of RNAi therapeutics is their sequence-based selectivity besides the dose dependency, making them theoretically adaptable to a wide range of disease-associated genes. As such, the use of siRNAs or other forms of RNAi agents offers a complementary strategy to act on therapeutic targets to achieve the control of diseases that are traditionally considered “undruggable” or more challenging by using small-molecule compounds or antibodies (Yu et al., 2020; Traber and Yu, 2024).

With an improved understanding of disease etiology and development of RNAi technologies, a number of siRNA drugs, including patisiran, givosiran, lumasiran, inclisiran, vutrisiran, nedosiran, fitusiran, and plozasiran, have been approved by the United States Food and Drug Administration (FDA), underscoring the translational potential of siRNAs and a broader RNAi-based therapies (Hu et al., 2020; Traber and Yu, 2023; Sehgal et al., 2024; Traber and Yu, 2024; Cronin and Yu, 2025). In addition to siRNAs, many other forms of RNAi agents are also under active development for various therapeutic indications. Despite these successes, most RNAi molecules rely heavily on chemical modifications and complex formulation strategies that facilitate RNA metabolic stability and efficient distribution or delivery to target tissues to achieve gene silencing and disease management. While extensive chemical modifications introduced through *in vitro* organic synthesis can lead to favorable pharmacokinetic properties needed for effective RNAi actions, they may also alter the natural structures of RNA molecules, and concerns have been raised about their safety, immunogenicity, and scalability (Yu et al., 2019; Traber and Yu, 2023; Sehgal et al., 2024).

To open up new avenues for the production of RNA molecules in live cells *in vivo*, our laboratory has developed an RNA molecular bioengineering platform technology, which utilizes specific tRNA-fused pre-miRNA carriers to achieve high-yield and large-scale *in vivo* production of target recombinant or bioengineered RNA (BioRNA) molecules bearing various forms of payload small RNAs, such as siRNAs, miRNAs, and aptamers (Chen et al., 2015; Ho et al., 2018; Li et al., 2021; Batra et al., 2024; Traber et al., 2024). BioRNAs purified from the host bacterial RNAs only contain a few natural posttranscriptional modifications and exhibit favorable stability once introduced into mammalian cells and animal models, enabling the selective release of payload siRNAs or miRNAs to exert specific RNAi actions and therapeutic potential (Chen et al., 2015; Li et al., 2015; Wang et al., 2015; Ho et al., 2018; Deng et al., 2021; Li et al., 2021; Chen et al., 2023; Batra et al., 2024; Traber et al., 2025). This RNA bioengineering technology provides an alternative means to current approaches, enabling *in vivo* production of target BioRNAs as a novel class of RNA molecules for both basic research and therapeutic development (Yu et al., 2019; Traber and Yu, 2023).

Delivery systems are critical for RNAs, including BioRNAs, to avoid the degradation by various RNases and achieve passing the target cell membranes to elicit RNAi actions. While lipid- and polymer-based transfection reagents such as the Lipofectamine are widely used for proof-of-concept studies, a high performance shown in cells *in vitro* does not necessarily predict *in vivo* efficacy due to complex physiological barriers (Wang et al., 2019). For *in vivo* applications, lipid nanoparticles (LNP) based delivery systems and advanced polymeric formulations are commonly used. LNP formulations typically incorporate cholesterol and pegylated lipids (PEG-lipids) as key structural components (Huang et al., 2016; Springer and Dowdy, 2018; Yu et al., 2020; Kalita et al., 2022; Song et al., 2024). Among them, polymeric systems such as *in vivo*-jetPEI, biodegradable poly (β-amino esters) (PBAEs) or poly (lactic-co-glycolic acid) (PLGA)-based nanoparticles have been developed to improve biocompatibility and controlled release (Xue et al., 2015; Kaczmarek et al., 2016). By contrast, LNPs that utilize ionizable lipids (e.g., DLin-MC3-DMA) are the most clinically validated platforms, including the formulation of siRNA drug patisiran and COVID-19 mRNA vaccines (Hou et al., 2021; Jung et al., 2022; Yu and Tu, 2022; An, 2024). Rather, an improved metabolic stability and excessive hepatic RNA exposure may lead to hepatotoxicity and unwanted immune activation (Hou et al., 2021; Wang et al., 2024). To enhance tissue- or cell-specific uptake, these platforms may be coupled with targeting ligands, among them the *N*-acetylgalactosamine (GalNAc) has proven success for hepatic delivery and is found in multiple FDA-approved RNAi drugs (Debacker et al., 2020; Zhang et al., 2022; Traber and Yu, 2023; Cronin and Yu, 2025).

Nevertheless, the translational performance of RNAi agents is strongly influenced by delivery efficiency and formulation-dependent safety profiles, underscoring the need for systematic evaluation of RNAi delivery platforms *in vivo*. The aim of this study was to evaluate and compare the efficiency and safety of five common *in vivo* nucleic acid delivery agents for systemic administration of BioRNA molecules. We chose a novel bioengineered green fluorescent protein (GFP) siRNA (BioRNA^Gly^/GFP-siRNA) as a model molecule (Batra et al., 2024) and critically compared the RNAi efficacy and safety profiles in GFP-transgenic mice, as formulated with five different *in vivo* delivery agents, including Invivofectamine® 3.0, a lipid-based reagent optimized for systemic RNA delivery; DLin-MC3-based LNP (used for patisiran); the LIPID-based *In Vivo* Transfection Reagent, a cationic liposome optimized for systemic and intratumoral delivery; the Nanoparticle-based *In Vivo* Transfection Reagent, a chemically engineered nanoparticle formulation; and the PEG-Liposome *In Vivo* Transfection Reagent, a PEGylated cationic liposome designed to reduce toxicity and inflammatory responses. Our results showed that, while both Invivofectamine and LNP formulations achieved greater degrees of hepatic siRNA accumulation and all products led to significant target gene silencing, none but the LNP product caused obvious liver toxicity and significant immune activation. In addition, the performance of these *in vivo* delivery agents in mice was not necessarily predictable by data obtained from cells *in vitro*.

## 2 Materials and methods

### 2.1 Chemicals and materials

The human glycyl tRNA fused pre-miR-34a carrier-based GFP-siRNA (BioRNA^Gly^/GFP-siRNA) and control RNA were generated through *in vivo* fermentation production and purified by anion exchange FPLC methods as recently described (Batra et al., 2024), yielding final RNA product with high homogeneity (>98% by HPLC) and low endotoxin levels (<5 EU/μg RNA). DMEM culture medium, phosphate-buffered saline (PBS), 0.05% trypsin-EDTA solution, fetal bovine serum (FBS), Opti-MEM medium and Lipofectamine 3000 transfection reagent were all obtained from Thermo Fisher Scientific (Waltham, MA, United States). TRIzol reagent was provided by Sigma-Aldrich (St. Louis, MO, United States). The Direct-zol RNA miniprep kit was obtained from Zymo Research (Irvine, CA, United States). Oligonucleotide primers were synthesized by Integrated DNA Technologies (Coralville, IA, United States). Isoflurane (USP grade, liquid for inhalation) was obtained from the Veterinary Medical Hospital Pharmacy at the University of California - Davis. Unless otherwise specified, additional laboratory chemicals and analytical-grade organic solvents were purchased from Thermo Fisher Scientific or Sigma-Aldrich.

### 2.2 Cell culture

The Huh7 cell line stably expressing GFP (Huh7-GFP) was established in our lab as described previously (Jilek et al., 2019). Cells were cultured in DMEM supplemented with 10% fetal bovine serum and maintained at 37 °C in a humidified incubator with 5% CO_2_.

### 2.3 BioRNA formulations

To prepare BioRNA^Gly^/GFP-siRNA injections, five *in vivo* delivery agents were chosen in this study. A custom lipid nanoparticle (LNP) formulation was synthesized by PackGene Biotech (Houston, TX, United States), using a DLin-MC3-based lipid composition (the same as Patisiran) to encapsulate BioRNA^Gly^/GFP-siRNA at a concentration of 0.5 mg/mL, and stored at −80 °C before use. Further, Invivofectamine® 3.0 (Thermo Fisher Scientific, Cat. No. IVF3001), a lipid-based *in vivo* transfection reagent optimized for systemic RNA delivery, was used to encapsulate BioRNA^Gly^/GFP-siRNA in the lab according to the manufacturer’s instructions. In addition, three other agents were obtained from Altogen Biosystems, including the LIPID-based *In Vivo* Transfection Reagent (Cat. No. 5011), a cationic liposome optimized for systemic and intratumoral delivery of small RNAs and plasmid DNA; the Nanoparticle-based *In Vivo* Transfection Reagent (Cat. No. 5031), a chemically engineered nanoparticle formulation suitable for siRNA, shRNA, miRNA, and plasmid DNA delivery; and the PEG-Liposome *In Vivo* Transfection Reagent (Cat. No. 5041), a PEGylated cationic liposome designed to reduce toxicity and inflammatory responses during systemic nucleic acid delivery. Each agent was used to prepare BioRNA^Gly^/GFP-siRNA formulations as instructed by the manufacturer. These complexes were freshly prepared prior to injection and administered via the tail vein to GFP-transgenic mice at the indicated doses. These formulations are abbreviated as LNP-BioRNA^Gly^/GFP-siRNA, Invivo-BioRNA^Gly^/GFP-siRNA, Nano-BioRNA^Gly^/GFP-siRNA, Lipid-BioRNA^Gly^/GFP-siRNA, and PEG-BioRNA^Gly^/GFP-siRNA, respectively.

### 2.4 *In vitro* knockdown of GFP

Huh7-GFP cells were seeded at a density of 5 × 10^4^ cells per well in 12-well plates. After 24 h, the cells were transfected with 15 nM of BioRNA^Gly^/GFP-siRNA or control RNA using the indicated delivery reagent, or the transfection reagent itself (vehicle), except the LNP formulation in which no LNP-BioRNA^Gly^/control RNA formulation was made and used, but the culture media mock transfection (vehicle) served as a control. In addition, Lipofectamine 3000 was included as a positive control in this study. Following the transfection, cells were maintained in an IncuCyte S3 Live-Cell Imaging System (Sartorius, Essen BioScience). GFP fluorescence intensity and cell confluency were monitored every 6 h for a total of 72 h using a ×4 objective lens.

### 2.5 *In vivo* knockdown of GFP

All animal procedures were reviewed and approved by the Institutional Animal Care and Use Committee (IACUC) at the University of California - Davis. Mice were maintained under specific pathogen-free conditions in individually ventilated cages at constant temperature and humidity, with free access to food and water. Animals were allowed to acclimate for 1 week prior to experimentation.

Six-to seven-week-old, male GFP-transgenic mice (C57BL/6-Tg (CAGEGFP)1Osb/J; The Jackson Laboratory, Bar Harbor, ME, United States) were administered 30 μg of BioRNA^Gly^/GFP-siRNA, formulated with five different *in vivo* delivery agents, via tail vein injection every other day for a total of three doses. Body weight was monitored closely throughout the study. As a negative control, an equal volume (200 μL) of PBS was injected following the same schedule (Blank group).

The day after the final injection, mice were anesthetized with isoflurane (3%–5%) delivered with oxygen in an induction chamber until loss of responsiveness was observed, followed by cervical dislocation for euthanasia. Major organs were then harvested for weight comparison. While GFP is ubiquitously expressed in transgenic mouse, the liver was selected as the primary organ for evaluating RNAi effects because it represents the dominant site for accumulation of systemically administered RNAs. To determine GFP fluorescence intensity, whole liver tissues were directly imaged using a ChemiDoc™ MP Imaging System (Bio-Rad, Hercules, CA, United States) under fluorescence mode to visualize GFP signals. Further, portions of the livers were embedded in Tissue-Tek O.C.T. compound (Sakura Finetek, Torrance, CA, United States), cryosectioned at 8 μm, and examined on a Leica Stellaris 5 confocal platform coupled with a Leica DMi8 inverted microscope and Leica Application Suite software (v4.4.0.24861; Leica Microsystems) using ×10 objectives.

### 2.6 Reverse-transcription, quantitative real-time polymerase chain reaction (RT-qPCR) analyses

Total RNA was extracted from cells or mouse liver tissues, and reverse-transcription quantitative PCR was performed as previously described to determine GFP siRNA and mRNA levels (Chen et al., 2015; Batra et al., 2024; Traber and Yu, 2024). All reactions were performed using iTaq Universal SYBR Green Supermix on a CFX96 Touch Real-Time PCR Detection System (Bio-Rad). Specifically, GFP siRNA was quantified by using specific stem-loop RT and qPCR primers, and qPCR analysis of GFP mRNA levels was conducted with gene-specific primers (Supplementary Table S1). U6 snRNA and 18S rRNA (primers summarized in Supplementary Table S1) were used as internal controls for normalization of GFP siRNA and mRNA levels, respectively, by using formula 2^−ΔΔC^T. Each treatment group was further compared with the vehicle or blank control group.

### 2.7 Safety profiles of different delivery systems

In addition, serum samples were collected from individual mice in the end of study for blood chemistry analysis (The Comparative Pathology Laboratory at University of California - Davis). This panel provided quantitation of key blood biochemistry indicators of hepatic and renal function, including alanine aminotransferase (ALT), aspartate aminotransferase (AST), albumin, alkaline phosphatase (ALP), blood urea nitrogen (BUN), creatinine, total bilirubin, and total proteins. Normal reference ranges for blood biochemistry markers, derived from healthy BALB/c mice, were provided by the testing laboratory (Traber et al., 2025). To further investigate the systemic immune responses, all samples were subjected to a multiplex bead-based cytokine immunoassay (Mouse Cytokine 32-Plex Discovery Assay® by Eve Technologies, Calgary, Canada). This high-throughput technology utilizes Luminex xMAP multi-analyte profiling, allowing for the simultaneous quantification of multiple distinct cytokines and chemokines such as IL-6, TNF-α, IFN-γ, and MCP-1-from a single serum aliquot.

### 2.8 Statistics

All values are expressed as mean ± standard deviation (SD). Statistical analyses were performed using one-way or two-way ANOVA followed by Bonferroni *post hoc* tests (GraphPad Prism, GraphPad Software). The probability value of *P* < 0.05 was considered statistically significant.

## 3 Results

### 3.1 GFP-siRNA levels in mouse liver tissues as delivered by five different agents

**FIGURE 1:**
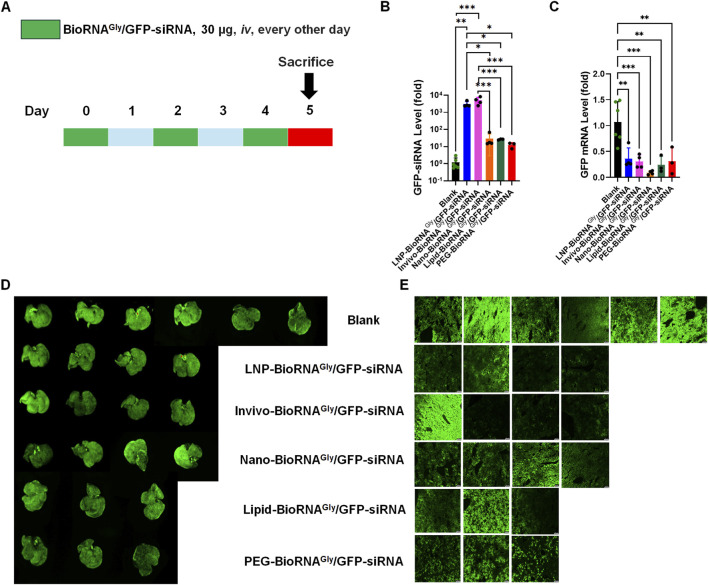
Comparative efficacy of BioRNA^Gly^/GFP-siRNA formulated with five different agents in GFP-transgenic mice *in vivo*. **(A)** Schematic representation of the dosing regimen. GFP-transgenic mice (C57BL/6-Tg (CAGEGFP)1Osb/J) were administered iv (via tail vein) with 30 μg of BioRNA^Gly^/GFP-siRNA every other day for three doses and then sacrificed on day 5, which were delivered in five different ways, including lipid nanoparticle (LNP) made by PackGene Biotech and Invivofectamine (Invivo) from Thermo Fisher Scientific, as well as a Nanoparticle (Nano), LIPID (lipid), and PEG-Liposome (PEG) based *In Vivo* Transfection Reagents from Altogen Biosystems. **(B)** GFP siRNA levels in mouse liver tissues were quantified by selective stem-loop RT-qPCR, indicating successful delivery and processing of BioRNA^Gly^/GFP-siRNA. Notably, GFP siRNA levels in the LNP and Invivofectamine groups were significantly higher than those in the other formulation groups. **(C)** GFP mRNA levels in mouse livers were determined by RT-qPCR, showing variable degrees of target gene silencing by different formulations. **(D)** *Ex vivo* whole-liver fluorescence and **(E)** frozen liver section imaging demonstrated the variations in suppressing GFP by different formulations. Overall, all formulations offered effective reductions in GFP mRNA levels and fluorescence intensities while the Invivofectamine and LNP formulations led to greater degrees of siRNA accumulation in the liver. Data are mean ± SD. **P < 0.01 and ***P < 0.001 (one-way ANOVA with Bonferroni *post hoc* tests). Blank: N = 6; LNP-BioRNA^Gly^/GFP-siRNA: N = 4; Invivo-BioRNA^Gly^/GFP-siRNA: N = 4; Nano-BioRNA^Gly^/GFP-siRNA: N = 4; Lipid-BioRNA^Gly^/GFP-siRNA: N = 3; PEG-BioRNA^Gly^/GFP-siRNA: N = 3.

To compare the *in vivo* delivery performance of selected delivery systems, we administered individual BioRNA^Gly^/GFP-siRNA formulations to GFP-transgenic mice via tail vein injection. The mice received 30 μg bioengineered siRNA every other day for a total of three doses (Figure 1A). Stem-loop RT-qPCR analysis of GFP-siRNA levels confirmed the effectiveness of each *in vivo* delivery agent, processing, and accumulation of the siRNA in mouse liver (Figure 1B). Interestingly, the mouse hepatic GFP-siRNA levels in the Invivo- and LNP-BioRNA^Gly^/GFP-siRNA treatment groups were about 100-fold higher than those delivered by the nanoparticle-, lipid-, and PEG-based agents (Figure 1B), indicating a more efficient delivery or subsequent release and accumulation of siRNA by the Invivofectamine and LNP systems.

### 3.2 Effectiveness of various BioRNA^Gly^/GFP-siRNA formulations to suppress hepatic GFP mRNA and fluorescence levels in transgenic mice

To define the RNAi effectiveness of various siRNA formulations, the GFP mRNA levels and fluorescence intensities in GFP-transgenic mouse liver tissues were examined and compared between different treatment groups. RT-qPCR analyses demonstrated a significant reduction in GFP mRNA levels across all treatment groups, as compared to the Blank control (Figure 1C). While all five siRNA formulations examined in this study demonstrated 70%–90% reduction of hepatic GFP mRNA levels, with some minor variations, the Nano-BioRNA^Gly^/GFP-siRNA offered relatively the greatest degree of mRNA knockdown. On the other hand, the excised livers were subjected to *ex vivo* imaging, and the results showed that, while Nano-, Lipid- and PEG-formulations caused modest degrees of reduction in GFP fluorescence, the Invivo- and LNP-BioRNA^Gly^/GFP-siRNA treatments led to more obvious decreases in GFP fluorescence when compared with the Blank control group, although there were considerable inter-animal variations (Figure 1D).

The efficacy to knockdown target gene expression by GFP-siRNA delivered by the five agents was also supported by fluorescence imaging of frozen liver sections (Figure 1E). Among them, LNP-, Invivo-, and Nano-BioRNA^Gly^/GFP-siRNA treatments exhibited a consistently effective reduction of GFP fluorescence signal, while Lipid- and PEG formulations led to relatively lower degrees of reduction. It is also noteworthy that the fluorescence intensities of whole livers and the corresponding tissue sections showed consistent results among individual animals and treatment groups. Collectively, our results demonstrate that BioRNA^Gly^/GFP-siRNA is efficiently delivered to livers by all five agents tested, in which the Invivofectamine and LNP offer the greater efficiencies in BioRNA^Gly^/GFP-siRNA delivery and hepatic target gene knockdown in mice *in vivo*.

### 3.3 Tolerability and safety profiles

**FIGURE 2:**
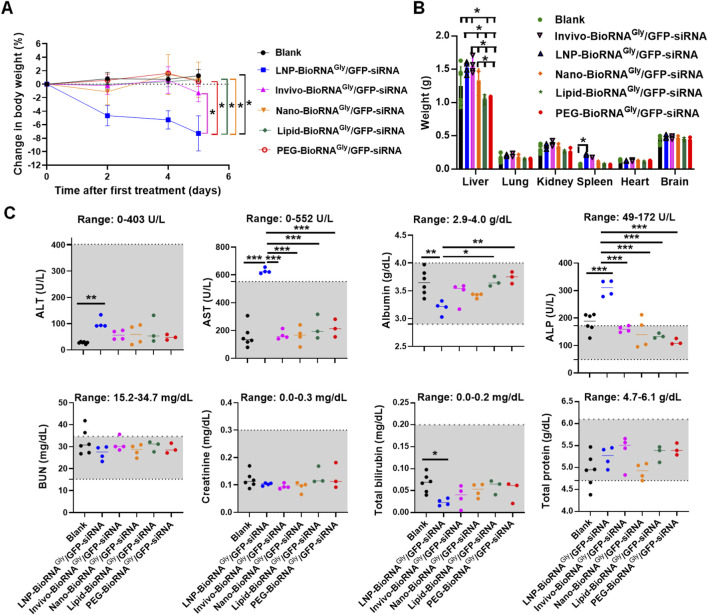
Safety profiles of various BioRNA^Gly^/GFP-siRNA formulations in GFP-transgenic mice. **(A)** Except an 8%, statistically significant decrease observed in the LNP group, mouse body weights showed no significant differences between any RNA treatment and blank control groups. **(B)** Weights of mouse major organs, including the lung, kidney, heart, and brain, were comparable between the RNA treatment and blank groups, while LNP product led to an obvious hepatosplenomegaly. Liver weights were also significantly increased by Invivofectamine formulation, while a modest enlargement of spleen is not statistically significant. **(C)** Mouse blood biochemistry profiles showed that LNP formulation altered the levels of a number of biomarkers, including ALT, AST, albumin, ALP, and total bilirubin, whereas other formulations had no effects. The reference ranges were derived from healthy BALB/c mice and provided by the Comparative Pathology Laboratory at University of California - Davis. Data are mean ± SD. *P < 0.05, **P < 0.01, and ***P < 0.001 (one- or two-way ANOVA with Bonferroni *post hoc* tests). Note that only statistically significant pairs are denoted herein, and other unmarked groups are not statistically significant (ns; P > 0.05).

The safety and tolerability profiles of these formulations were further compared by examining serum biochemistries besides monitoring mouse body weights and excised organ weights. The MC-3-based LNP formulation was associated with a modest (around 8%) decrease in mouse body weights during the study, and it is statistically significant (P < 0.05) when compared to Blank control or any other formulation (Figure 2A). By contrast, mouse body weights remained steady in all other formulation treatment groups, similar as the Blank control. Furthermore, the weights of major organs such as kidney, lung, heart, and brain did not differ among all treatment groups (Figure 2B; Supplementary Figure S1). However, liver and spleen exhibited some changes after treatments. Specifically, liver weights were significantly (P < 0.05) increased in the LNP- (1.52 ± 0.09 g) and Invivo-BioRNA^Gly^/GFP-siRNA (1.55 ± 0.12 g) groups, as compared with the Blank (1.25 ± 0.29 g) and other treatments groups, while the Lipid (1.05 ± 0.07 g) and PEG (1.07 ± 0.02 g) groups showed slightly lower liver weights than the Blank control and other groups (Nano-formulation: 1.34 ± 0.17 g) but statistically significant (P < 0.05). Moreover, the LNP product led to a statistically significant (P < 0.05) enlargement of spleen tissues (0.23 ± 0.01 g) when compared to Blank control (0.07 ± 0.02 g) (Figure 2B), indicating potential immune activation or splenic stress. Though the spleen weights were obviously greater in the Invivo-BioRNA^Gly^/GFP-siRNA group (0.18 ± 0.01 g), it is not statistically significant (P = 0.52) when compared with the Blank control group. By contrast, spleen sizes in all other treatment groups (Nano-, Lipid-, and PEG-formulations: 0.10 ± 0.02 g, 0.07 ± 0.02 g, and 0.07 ± 0.01 g, respectively) were comparable as the control treatment (Figure 2B).

In addition, blood biochemistry analyses revealed an obvious alteration by the LNP formulated BioRNA treatment, whereas all other formulation groups showed similar profiles as the Blank control group (Figure 2C). In particular, the ALT, AST and ALP levels were elevated in the LNP group (P < 0.01), and the albumin and total bilirubin levels were reduced (P < 0.05), demonstrating the perturbation of regular hepatic and renal functions. In addition, higher levels of AST and ALP in the LNP group are also statistically significant (P < 0.001) when compared with all other BioRNA formulations (Figure 2C). Taken together, these results indicate that Invivofectamine, nanoparticle-, lipid- and PEG-based BioRNA^Gly^/GFP-siRNA formulations were generally well tolerated in mice, whereas the MC3-based LNP product obviously caused modest toxicities, as manifested by the reduction of mouse body weight and hepatosplenomegaly, as well as alterations of liver and kidney function biomarkers.

### 3.4 Perturbation of mouse immunity

**FIGURE 3:**
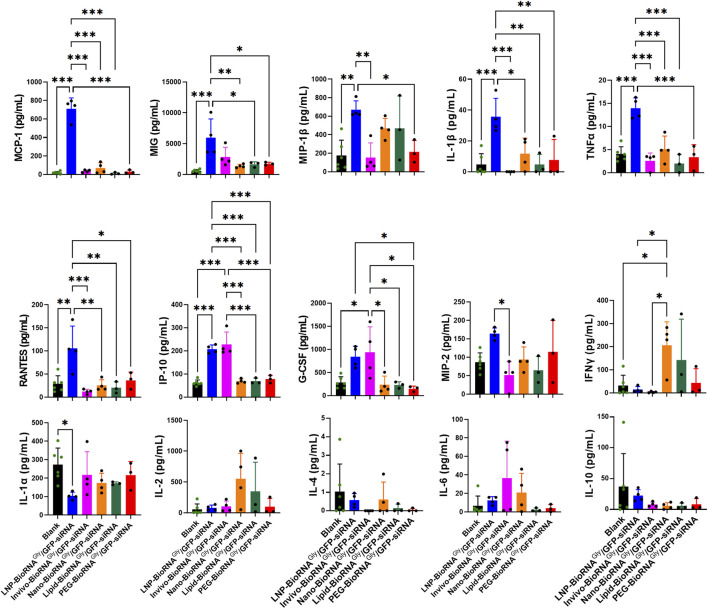
Influence of different BioRNA^Gly^/GFP-siRNA formulations on immune responses in GFP-transgenic mice, as manifested by a panel of serum cytokine profiles determined by a Multiplex Cytokine Assay (Eve Technologies). Overall, the LNP formulation remarkably elevated many pro-inflammatory mediators, including MCP-1 (CCL2), MIG (CXCL9), MIP-1β (CCL4), IL-1β, TNFα, RANTES (CCL5), IP-10 (CXCL10), G-CSF, and MIP-2 (CXCL2), compared with the Blank group, indicating strong innate immune activation. In contrast, anti-inflammatory cytokines, including IL-4 and IL-10, did not show any statistically significant differences among treatment groups. Meanwhile, the Invivofectamine and nanoparticle formulations caused mild changes in particular cytokines (e.g., increase in IFNγ by Nano-BioRNA product; and increase in IP-10 and G-CSF by Invivo-BioRNA product), whereas the Lipid and PEG formulations had no or minimal effects. Data are mean ± SD. *P < 0.05, **P < 0.01, and ***P < 0.001 (one-way ANOVA with Bonferroni *post hoc* tests). Note that only statistically significant pairs are denoted while other unmarked groups are not statistically significant (ns; P > 0.05).

To determine and compare the degrees to which individual formulations would induce immune responses, the levels of a panel of 32 mouse cytokines in the blood samples were measured (Figure 3; Supplementary Figure S2). Overall, mice treated with the LNP-BioRNA product displayed notable changes in many cytokines. Compared to the Blank control, LNP formulation largely elevated many pro-inflammatory cytokines, such as MCP-1/CCL2 (P < 0.001), MIG/CXCL9 (P < 0.001), MIP-1β/CCL4 (P < 0.01), IL-1β (P < 0.001), TNFα (P < 0.001), RANTES/CCL5 (P < 0.01), and IP-10/CXCL10 (P < 0.001). Among them, MCP-1 levels were increased to the greatest degree (∼37-fold), followed by MIG (∼12-fold) and IL-1β (∼7-fold). By contrast, Invivofectamine and nanoparticle formulations only induced some changes in one or two cytokines (i.e., increase in IP-10 and G-CSF levels by Invivo-BioRNA; and increase in IFNγ levels by Nano-BioRNA), and the Lipid- and PEG-BioRNA products had no or minimal effects on these cytokines, when compared with the Blank control group. These results demonstrate that, compared to other *in vivo* delivery agents studied herein, LNP formulation poses a high risk of inducing immune responses or severe cytokine release syndrome.

### 3.5 *In vitro* transfection and GFP knockdown efficiency of BioRNA^Gly^/GFP-siRNA delivered with different agents

**FIGURE 4:**
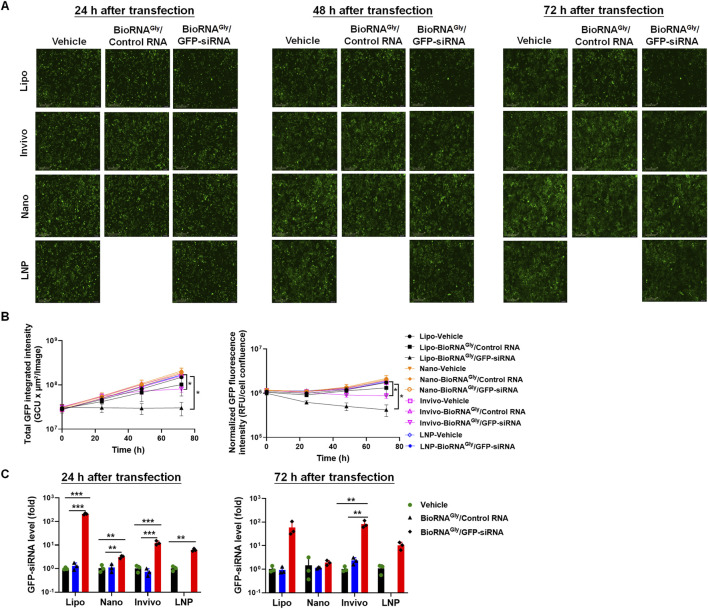
Comparative efficiency of various BioRNA^Gly^/GFP-siRNA formulations to reduce GFP intensities in GFP-expressing Huh7 cells *in vitro*. Cells were transfected with 15 nM of BioRNA^Gly^/GFP-siRNA, control RNA, or vehicle using the indicated reagents, with Lipofectamine 3000 (Lipo) as a positive control. **(A)** Representative GFP fluorescence images at 72 h post-transfection. The Invivofectamine (*Invivo*) formulated siRNA significantly reduced GFP fluorescence intensity at 72 h post-transfection, whereas nanoparticle (Nano) and LNP formulated siRNAs did not show much suppression of GFP fluorescence intensities, when compared with control RNA or vehicle. **(B)** Quantification of GFP fluorescence intensity. Absolute fluorescence intensities (left) and those (right) normalized to respective cell confluency measured by IncuCyte live-cell imaging. **(C)** Changes in intracellular GFP-siRNA levels in different treatment groups as determined by selective stem-loop RT-qPCR assay. Note that LNP-BioRNA^Gly^/Control RNA formulation was not prepared for this study, and thus there are no related data. Data are presented as mean ± SD, n = 3. *P < 0.05, **P < 0.01, ***P < 0.001 (one- or two-way ANOVA with Bonferroni *post hoc* tests). Lipo: Lipofectamine 3000 transfection reagent. Other unmarked groups are not statistically significant (ns; P > 0.05).

Given the findings on *in vivo* knockdown efficiency, we chose three GFP-siRNA formulations, namely LNP, Invivofectamine, and nanoparticles, to determine their *in vitro* efficiency in Huh7-GFP cells and to compare the outcomes between *in vivo* and *in vitro* models. After transfection, cells were subjected to IncuCyte live-cell imaging study, and cells were monitored for 72 h. As a positive control, the broadly used, *in vitro* transfection agent Lipofectamine (Lipo) product offered the greatest degree of reduction of GFP fluorescence (∼80% at 72 h; Figures 4A,B), when compared with either control RNA or vehicle treatment, which was associated with high levels of intracellular GFP-siRNA persisting over 72 h (Figure 4C). By contrast, the three *in vivo* transfection agents did not or just modestly suppressed GFP signals in cells (Figures 4A–C). In particular, when compared with the respective control RNA or vehicle treatment, Invivofectamine formulation reduced GFP fluorescence by ∼50% at 72 h, accompanied by high levels of GFP-siRNA within cells. The nanoparticle and LNP products yielded marginal (<10%) reduction of GFP fluorescence at 72 h, where the LNP group showed some intracellular accumulation of GFP-siRNA, and nanoparticle treatment showed minimal. These findings suggest that the transfection and knockdown efficiency of *in vivo* transfection agents may not be predictable by results obtained from *in vitro* models.

## 4 Discussion

In this study, we compared side-by-side the efficiency and safety of five common *in vivo* delivery systems, including LNP, lipid carriers, PEG-liposomes, nanoparticles, and Invivofectamine, for the delivery of novel bioengineered RNAi reagents where the BioRNA^Gly^/GFP-siRNA served as a model molecule to knock down hepatic GFP in transgenic mice. All formulations led to siRNA accumulation in mouse liver tissues and effectively reduced hepatic GFP expression *in vivo*, among them the LNP, Invivofectamine and nanoparticles achieved greater RNAi effects. While the BioRNA-encapsulated Invivofectamine, nanoparticle, lipid, and PEG-liposome products were all well tolerated, the MC3-based LNP formulation was associated with loss of mouse body weights, enlargement of liver and spleen tissues, and changes in liver and kidney function markers as well as multiple cytokines. Further, except that Invivofectamine exhibited effective siRNA delivery and target gene silencing in cells *in vitro*, results obtained from *in vitro* models for these *in vivo* delivery agents might not be used for the prediction of *in vivo* effectiveness. The differences of these delivery agents established herein highlight the importance of assessing both efficacy and safety towards identifying proper delivery platforms for RNA therapeutics.

The LNPs, typically composed of ionizable and PEGylated lipids as well as cholesterol, represent the most commonly used systems for *in vivo* delivery of therapeutic and immunogenic RNAs, such as siRNAs, mRNAs, and other forms of RNAs. LNP agents have been successfully translated into clinical applications, including the siRNA drug patisiran and COVID-19 mRNA vaccines (Yu et al., 2020; Hou et al., 2021; Yu and Tu, 2022; Traber and Yu, 2024). However, concerns remain about the safety of RNA therapeutics- or vaccine-loaded LNP products, especially the risk of immune activation or even severe cytokine release storm (Yonezawa et al., 2020; Yu et al., 2020; Verbeke et al., 2022; Traber and Yu, 2023; Wang et al., 2024). Indeed, there are growing clinical evidence, including those collected by the manufacturers, revealing a higher risk of serious adverse events, particularly the anaphylaxis, myocarditis, and pericarditis, for people administered LNP-mRNA vaccines (Fraiman et al., 2022; Patone et al., 2022; Yasuhara et al., 2023; Urdaneta et al., 2024), though it is obscure whether the toxicities are caused specifically by the LNP or mRNA or together. The systemic toxicities of the LNP-BioRNA product disclosed in this comparative study in mouse models, which are actually absent from the same BioRNA molecule formulated with four other agents, echo the concerns about safety of RNA pharmaceuticals-loaded LNP products. As the subjects (mice vs*.* humans), payloads (BioRNA vs*.* mRNA) and its dosing regimens (30 μg/mouse, intravenous, 3 doses in 1 week vs*.* 30–100 μg/person, intramuscular, two doses in 3–8 weeks (Polack et al., 2020; Baden et al., 2021)) as well as the LNP compositions in the present study vs*.* clinical research on COVID-19 vaccines differ largely, caution should be advised to directly connect those toxicities. It is also noteworthy that the LNP-BioRNA did not induce serum IFNγ levels in the present study as the LNP-loaded mRNA vaccine reported very recently (Cao et al., 2025), while both the present study and previous study (Cao et al., 2025) observed the elevation of serum IP-10 (CXCL10). This might be attributable to the differences in payload RNAs, LNP compositions, or mouse strains between that (Cao et al., 2025) and current studies, which reiterates caution to extrapolate preclinical observations.

The commercially available Invivofectamine agent developed for systemic delivery of RNAi agents has been used in some small animal studies (Inaba et al., 2012; Wang et al., 2013), yet its performance in comparison with other commercial agents remains unknown. The present study represents an independent and thorough evaluation of Invivofectamine with four other commercially available *in vivo* delivery agents by using novel BioRNA/siRNA molecules obtained through *in vivo* fermentation production (Li et al., 2021; Batra et al., 2024; Traber et al., 2024), distinguished from conventional RNA molecules made *in vitro* by chemical synthesis (e.g., siRNAs and miRNA mimics) or enzymatic reactions (e.g., mRNAs). It is noteworthy that single-stranded BioRNAs, approximately 180 nt in length, forming highly structured hairpins (Mao et al., 2024) and behaving like double-stranded RNAs, are presumably easier for formulation and delivery (Luo et al., 2024). By contrast, double-stranded siRNAs are shorter (20–22 bp) and might be leaky in the formulations, while single-stranded mRNAs are much longer (usually over 1,000 nt) and thus hard to bundle up. Anyhow, our findings on the delivery efficiency and safety profiles for Invivofectamine as compared with four other commercial agents provide a framework for investigators to select appropriate materials for preclinical RNAi investigations.

The divergent performances of bioengineered siRNA formulated with different agents are probably attributable to their distinct physicochemical characteristics and delivery mechanisms (Paunovska et al., 2022; Catenacci et al., 2024). LNPs, while highly efficient in packaging and protecting siRNAs with ionizable lipids and helper components, can trigger hepatotoxicity and systemic immune activation (Yonezawa et al., 2020; Verbeke et al., 2022), as identified in our study on the LNP with particular components and under certain experimental conditions. PEG-modification has been used to improve stability and duration, yet it may also reduce intracellular uptake and result in moderate RNAi effects (Majzoub et al., 2014; Hattori et al., 2019). As revealed in the present study, while providing high efficiency in RNA delivery and target gene silencing, the LNP product was the only formulation in this study that significantly elevated multiple pro-inflammatory cytokines in mice. And many of the elevated cytokines, such as MCP-1/CCL2, MIG/CXCL9, MIP-1β/CCL4, RANTES/CCL5, and IP-10/CXCL10, function to recruit monocytes, macrophages, neutrophils, and activated T cells (Choi et al., 2007; Deshmane et al., 2009; Marques et al., 2013; Tokunaga et al., 2018). An increase in IL-1β and TNFα indicates the activation of inflammatory response (Dinarello, 2018; Kany et al., 2019), and a higher G-CSF level suggests the stimulation of myeloid cell production (Richards et al., 2003; Park et al., 2022). IL-1α levels appeared lower than the Blank group in several treatment conditions. IL-1α differs biologically from IL-1β and is commonly described as a constitutively expressed, cell-associated alarmin rather than a classic inflammasome-dependent secreted cytokine (Chen et al., 2007; Rider et al., 2017; Dinarello, 2018). Therefore, this trend should be interpreted cautiously and does not necessarily indicate a reduction of inflammation. Collectively, these changes indicate an acute innate immune activation and leukocyte recruitment associated with the tested LNP formulation in this mouse model. Interestingly, nanoparticle-BioRNA product selectively induced the levels of IFNγ that has dual functions, pro-inflammatory and immunosuppressive, critical for cell defenses and implacable to various diseases including cancer (Ivashkiv, 2018).

It is also notable that the nanoparticle-formulated siRNA reduced GFP mRNA levels to the greatest extent, albeit a modest siRNA accumulation found in the liver that is lower than LNP and Invivofectamine products. While quantitative measurement of liver siRNA levels is informative, the lack of precise quantification of siRNAs within target hepatocytes may not necessarily indicate an “*in vivo* transfection efficiency”. Further, siRNA levels determined at a single time point may not be indicative of the cumulative RNAi outcomes evaluated at the same time point. Differences in siRNA intracellular trafficking and endosomal escape, as well as the release of functional antisense strand and RISC loading, among delivery systems may result in formulation-dependent silencing efficiency (Sahay et al., 2010; Gilleron et al., 2013). As such, the levels of siRNA within the RISC or cytoplasm would probably be more relevant to the RNAi efficacy. Furthermore, although hepatic GFP fluorescence intensities were reduced by individual siRNA formulations, the degrees of reduction in GFP fluorescence were not exactly proportional to the extents of GFP mRNA reduction. It should be noted that GFP-based fluorescence imaging may be influenced by intrinsic autofluorescence, including liver tissues. Therefore, fluorescent images in this study, exhibiting large inter-individual variations among a limited number of animals, were interpreted rather very cautiously and complemented by more quantitative and reliable mRNA measurements which, the latter, not only addresses siRNA mechanistic actions but also ensures rigorous assessment of RNAi efficacy. Actually, similar discrepancies between mRNA knockdown and protein-level readouts have been widely reported for stable reporter proteins (Tsien, 1998; Elbashir et al., 2001; Jackson et al., 2003). This is likely attributed to the high stability and apparently long half-life of GFP, presumably those pre-existing proteins that persist after an effective RNAi-mediated transcript knockdown. Indeed, previous studies have demonstrated that GFP exhibits remarkable intracellular stability, with reported half-life values more than 24 h in mammalian cells (Tsien, 1998; Corish and Tyler-Smith, 1999). By contrast, the half-life of pro-protein convertase subtilisin/kexin type 9 (PCSK9) protein, whose transcript is the target of a recently approved siRNA drug inclisiran (Traber and Yu, 2024; Cronin and Yu, 2025), is about 5 min in mice (Horton et al., 2009). Therefore, one would have a thorough understanding of RNAi effects by employing precise and accurate quantitative methods to examine siRNA as well as target mRNA and protein levels, and RNAi therapy should be directed to those mRNA targets whose encoded proteins possess shorter half-lives.

Additionally, most materials designed for *in vivo* delivery may not exhibit comparable effects in an *in vitro* setting. Our study showed that, although highly effective in mice *in vivo*, the LNP formulation showed minimal effects in cells *in vitro*. This divergence likely reflects fundamental differences in delivery mechanisms, in which the LNPs rely on systemic stability and hepatocyte-directed targeting in animals (Hou et al., 2021; Jung et al., 2022), but exhibit a limited level of cellular uptake and endosomal escape *in vitro* (Paunovska et al., 2018; Johansson et al., 2025). Furthermore, it is unknown whether the *in vitro* and *in vivo* dosages would be equivalent to yield comparable drug exposure to achieve similar cumulative pharmacodynamic effects. On the other hand, the use of Invivofectamine led to relatively consistent delivery efficiency and RNAi effects in mouse liver tissues and Huh7 cells. Nevertheless, caution is advised to use *in vitro* data to predict the *in vivo* performance of any *in vivo* transfection agents.

In conclusion, all five *in vivo* transfection agents examined in the present study offered efficient delivery of bioengineered siRNA molecules into mouse liver tissues *in vivo* to afford RNAi efficacy. However, the MC3-based LNP formulation, under the described experimental conditions, was associated with statistically significant changes in multiple outcomes as reported herein, whereas all other products were well tolerated. Highlighting the importance of balancing efficacy and safety in RNAi research and drug development, these findings on the differences among these commercially available *in vivo* delivery agents should offer clues to select proper systems for *in vivo* delivery of novel BioRNA molecules to achieve effective and safe therapy or vaccination.

## Data Availability

The original contributions presented in the study are included in the article/Supplementary Material, further inquiries can be directed to the corresponding author.
