# Supplementary material for: Efficiency and safety of five different agents for in vivo delivery of novel bioengineered RNAi molecules
Source: Front Mol Biosci. 2026 Mar 13;13:1785592. doi: 10.3389/fmolb.2026.1785592 (PMC13021479; doi:10.3389/fmolb.2026.1785592)

Supplemental Materials to

Efficiency and safety of five different agents for *in vivo* delivery of novel bioengineered RNAi molecules

Su Guan, Mei-Juan Tu, Yan-Ju Li, Yimei Wang, and Ai-Ming Yu*

Department of Biochemistry and Molecular Medicine, University of California at Davis, School of Medicine, Sacramento, CA 95817

Supplemental Table S1
Supplemental Figure S1-2

Supplementary Table S1. Sequences of individual primers used for stem-loop RT-qPCR and RT-qPCR analyses.

**Target**		**Primer Sequence (5' to 3')**
GFP-siRNA	RT	GTCGTATCCAGTGCAGGGTCCGAGGTATTCGCACTGGATACGACGGGCAC
	Forward	GCGCGCAGTTGTACTCCAGCTT
	Reverse	GTGCAGGGTCCGAGGT
U6	Forward	CTCGCTTCGGCAGCACA
	Reverse	AACGCTTCACGAATTTGCGT
GFP-mRNA	Forward	ACGTAAACGGCCACAAGTTC
	Reverse	AAGTCGTGCTGCTTCATGTG
18S	Forward	AAGTCCCTGCCCTTTGTACACA
	Reverse	GATCCGAGGGCCTCACTAAAC

Figure S1. Organ weights as normalized to mouse body weights, following treatment with different BioRNA formulations. Data are calculated as percentages of corresponding body weights. Overall, the normalized organ weight results are consistent with the trends observed in absolute organ weights shown in Figure 2B. Amid large inter-animal variations, change in liver weights is statistically significant for the LNP-BioRNA and Invivo-BioRNA groups compared with the Blank control, as well as the Lipid-BioRNA and PEG-BioRNA groups. Spleen enlargement is in the LNP-BioRNA group is statistically significant compared with blank control. Lung, kidney, heart, and brain weights remained largely comparable across all treatment groups. Data are mean ± SD with individual data points shown. Statistical analysis was conducted using one-way ANOVA followed by Bonferroni *post hoc* multiple comparisons test across all groups. Note that only statistically significant pairs (*P < 0.05) are denoted, while other unmarked groups are not statistically significant (ns; P>0.05).

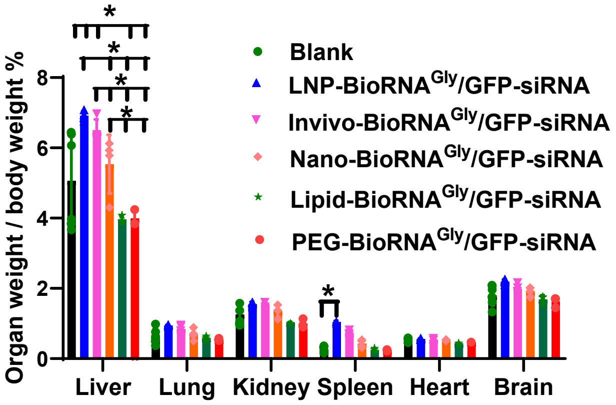

Figure S2. Additional cytokines examined in this study. These cytokines in mouse blood samples showed no significant changes by any BioRNA formulations, as compared with Blank group. Neither are there any statistically significant changes noted between different formulations. Note that none of these cytokine is statistically significant (ns; P>0.05) between any formulation and Blank control or between two different formulations.

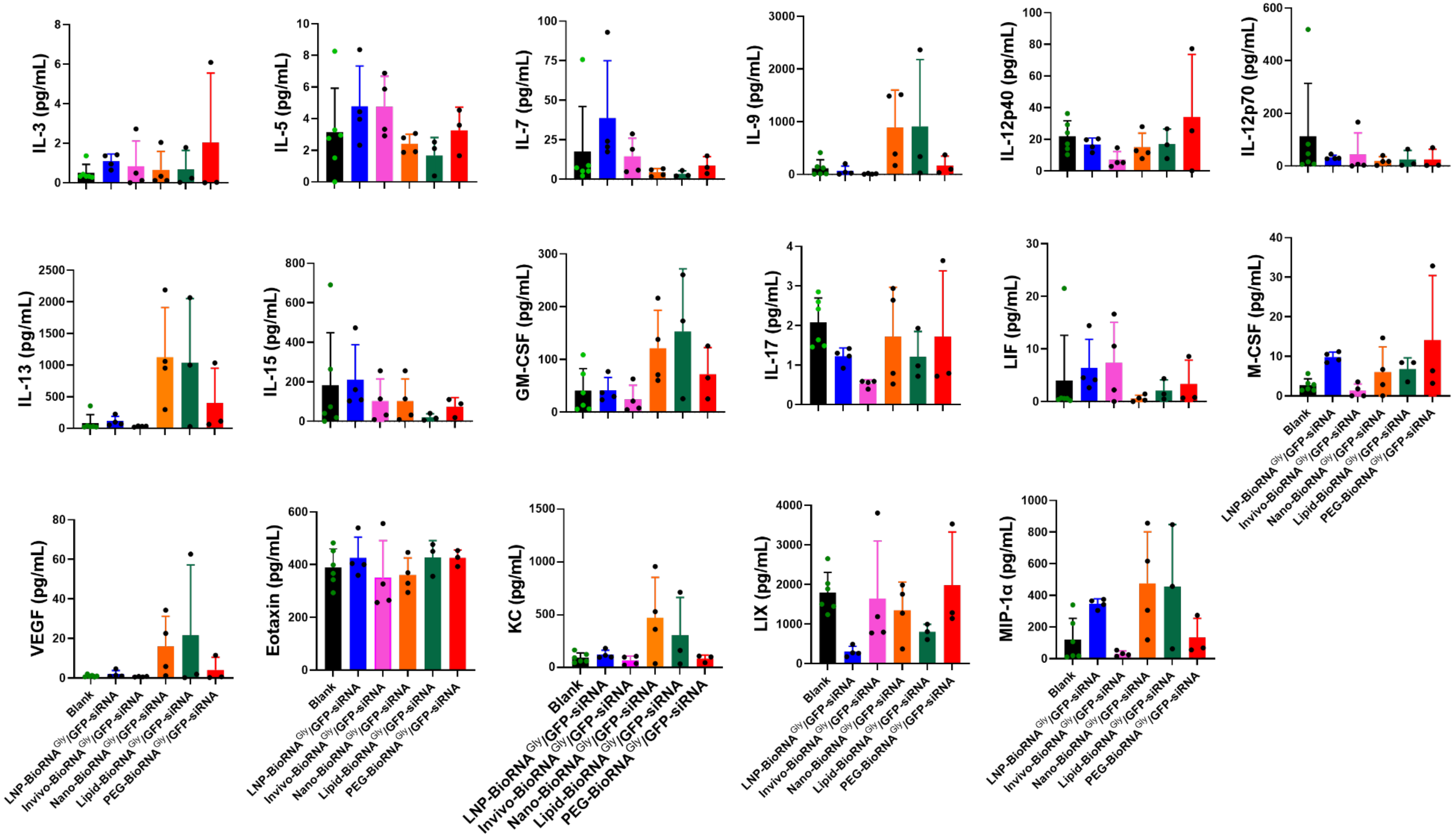

Supplement: Supplementary file 1 [file DataSheet1.pdf]
